# Isolation and Full-Genome Characterization of Nipah Viruses from Bats, Bangladesh

**DOI:** 10.3201/eid2501.180267

**Published:** 2019-01

**Authors:** Danielle E. Anderson, Ariful Islam, Gary Crameri, Shawn Todd, Ausraful Islam, Salah U. Khan, Adam Foord, Mohammed Z. Rahman, Ian H. Mendenhall, Stephen P. Luby, Emily S. Gurley, Peter Daszak, Jonathan H. Epstein, Lin-Fa Wang

**Affiliations:** Duke–National University of Singapore Medical School, Singapore (D.E. Anderson, I.H. Mendenhall, L.-F. Wang);; EcoHealth Alliance, New York, New York, USA (Ariful Islam, P. Daszak, J.H. Epstein);; CSIRO Australian Animal Health Laboratory, Geelong, Victoria, Australia (G. Crameri, S. Todd, A. Foord);; icddrb, Dhaka, Bangladesh (Ausraful Islam, M.Z. Rahman, E.S. Gurley); University of Guelph, Guelph, Ontario, Canada (S.U. Khan);; Stanford University, Stanford, California, USA (S.P. Luby);; Johns Hopkins Bloomberg School of Public Health, Baltimore, Maryland, USA (E.S. Gurley)

**Keywords:** henipavirus, Nipah virus, viruses, bats, Pteropus medius, isolation, next-generation sequencing, full-genome characterization, enrichment, zoonoses, Bangladesh

## Abstract

Despite molecular and serologic evidence of Nipah virus in bats from various locations, attempts to isolate live virus have been largely unsuccessful. We report isolation and full-genome characterization of 10 Nipah virus isolates from *Pteropus medius* bats sampled in Bangladesh during 2013 and 2014.

Nipah virus (NiV) is an emerging zoonotic virus carried by bats. It is considered a global health priority by the World Health Organization and has pandemic potential because of its zoonotic nature, human-to-human transmissibility, wide geographic distribution of bat reservoir species, high case-fatality rate in humans, and lack of available vaccine or therapeutic agents (*1*). Although NiV or NiV-related infections have been demonstrated by serologic surveillance or PCR detection in several bat species across extensive areas, attempts to isolate live NiV have been unsuccessful; there have been only 3 successful reports: *Pteropus hypomelanus* bats (*2*) and *P. vapmyrus* bats (*3*) in Malaysia and *P. lylei* bats in Cambodia (*4*).

Bangladesh has reported seasonal outbreaks of infectious NiV almost annually since 2001, and India has reported 2 outbreaks in neighboring West Bengal, the last in 2007 (*5*,*6*). In May 2018, India reported an outbreak in Kerala State, which is >1,800 km southwest of West Bengal (https://www.searo.who.int/entity/emerging_diseases/links/nipah_virus_outbreaks_sear/en/).

Despite sustained efforts to detect NiV in bats in this region, current infection data are largely from serologic and limited PCR detection of virus RNA from potential bat reservoirs, such as *P. medius* from Bangladesh and India (*7*–*10*). NiV genomic data from the region have come primarily from human cases (*11*,*12*). Spillover might not be limited to humans in Bangladesh; nonneutralizing antibodies against NiV in cattle, goats, and pigs (*13*) underscore the urgency of characterizing diversity of henipaviruses in *P. medius* bats and other possible animal reservoirs in the region.

We report isolation of NiVs from *P. medius* bats in Bangladesh. We performed full-genome characterization of these viruses by using enrichment-based next-generation sequencing (NGS).

## The Study

During January 2011–April 2014, we collected 2,749 bat samples from various ongoing projects in the region. We collected samples nondestructively from individual bats as described (*7*) and collected environmental urine samples from underneath roosts by using polyethylene sheets. In early 2013, an outbreak of infection with NiV occurred in 13 districts of Bangladesh (Gaibandha, Jhinaidaha, Kurigram, Kushtia, Magura, Manikganj, Mymenshingh, Naogaon, Natore, Nilphamari, Pabna, Rajbari, and Rajshahi). In April 2013, we collected only urine samples during an outbreak investigation in Raipur, Manikganj. We also tested 944 underroost urine samples, 829 throat swab specimens, and 976 urine samples from 2 confirmed bat species (*P. medius* and *Rousettus leschenaultia*) for NiV. Samples were collected with permission from the Government of Bangladesh Forestry Office and under Institutional Animal Care and Use Committees (Tufts University no. G2011-12 and University of California at Davis no. 19300).

**Figure 1:**
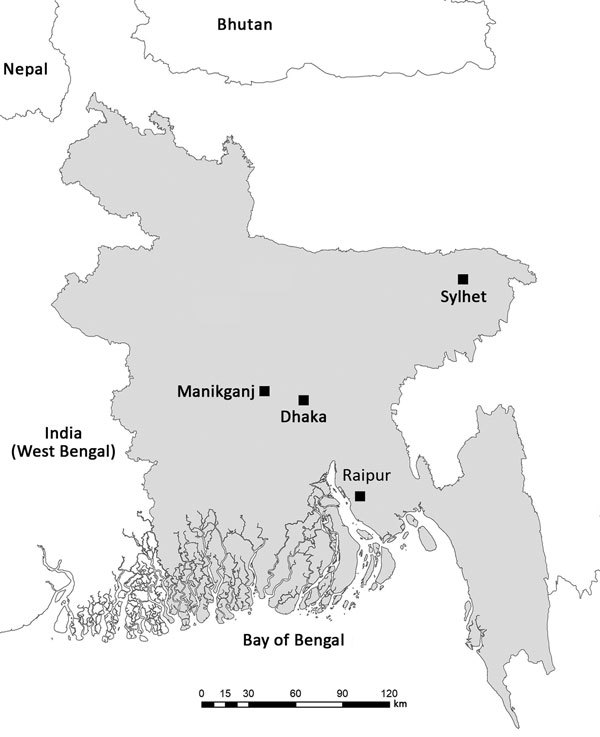
Sampling sites (Raipur, Manikganj, and Sylhet) for bat reservoirs of Nipah virus and location of the capital, Dhaka, in Bangladesh. Map was generated by using ArcGIS version 10.4.1 software (https://desktop.arcgis.com/en/quick-start-guides/10.4/arcgis-desktop-quick-start-guide.htm).

We also collected bat samples from 5 sites in Raipur that showed NiV spillover and 1 district (Sylhet) that had no reported cases (Figure 1). Samples were tested at the CSIRO Australian Animal Health Laboratory under BioSafety Level 4 containment. Individual samples were pooled into groups of 4 for initial extraction and PCR analysis. Of 688 pooled samples tested, 20 pools were positive by a nucleoprotein gene–specific 1-step reverse transcription quantitative PCR. The 80 individual samples from positive pools were thawed for virus isolation and RNA reextracted for PCR. Of 80 samples (urine and throat swab specimens) tested by nucleoprotein gene–gene specific 1-step reverse transcription quantitative PCR, only 20 urine samples were positive. We used these 20 urine samples for virus isolation.

We performed virus isolation under BioSafety Level 4 containment. For virus isolation from PCR-positive samples, we prepared Vero cells in 96-well plates in Eagle minimum essential medium (EMEM) containing 10% fetal bovine serum and 1× antibiotic–antimycotic mixture (GIBCO, http://www.biosciences.ie/gibco). We added 50 μL of each sample to 2 wells, incubated each sample for 90 min, remove the inoculum, and added 200 μL EMEM to each well. Plates were incubated at 37°C, checked at 7 days postinfection for a cytopathic effect (CPE), and frozen at −80°C.

A total of 11 wells showed CPE, and putative virus culture from these wells was passaged a second time by inoculation of a 24-well plate containing 80% confluent Vero cells in EMEM with 80 μL of culture supernatant from each positive well. Plates were incubated at 37°C for 90 min; inoculum was removed and 1 mL EMEM added. Plates were then incubated at 37°C for 5 days. All 11 samples remained CPE positive after this second passage.

For a third passage, we harvested culture supernatant and added 300 μL to a 25-cm flask containing 80% confluent Vero cells. Flasks were incubated for 90 min at 37°C before inoculum was removed and 5 mL EMEM added. We incubated the flasks at 37°C and harvested virus supernatants at 2–3 days postinfection. Supernatants were clarified by centrifugation at 10,000 × *g* for 5 min and frozen at −80°C.

To confirm the identity of isolated viruses, we subjected each sample to electron microscopy and PCR. All isolates had morphology consistent with NiV and were positive by NiV-specific PCR. Virus stocks were amplified in Vero cells, and supernatants were harvested and clarified by centrifugation at 10,000 × *g* for 10 min, followed by pelleting virus from supernatant by centrifugation at 200,000 × *g* for 30 min. Virus pellets were resuspended in 200 μL of Magmax Buffer (Applied Biosystems, https://www.thermofisher.com/us/en/home/brands/applied-biosystems.html) for RNA extraction according to the manufacturer’s protocol.

We used a virus enrichment strategy (*14*) to obtain the full-genome sequence for NiV isolates. Total RNA was used to prepare NGS libraries (New England Biolabs, https://www.neb.com/) according to the manufacturer’s instructions. DNA libraries were subjected to a liquid-based target capture method to separate and enrich for specific NiV sequences. NiV-specific 120-mer biotinylated DNA baits were designed to capture the entire genome. Hybridized probes and captured NiV genetic material were immobilized on magnetic beads, and contaminating host material was washed away, which increased the number of virus-specific reads. We mapped all reads to NiV sequence JN808863 by using the Map to Reference Function in Geneious version 7.1.6 (https://www.geneious.com/previous-versions/). The consensus nucleotide sequence was selected by majority rules at each site.

To ascertain 5′ and 3′ ends because of low coverage and PCR errors and chimeras, we downloaded full-genome NiV sequences from GenBank and aligned them by using MAFFT in Geneious. A 90% similarity threshold was used to generate consensus 5′ and 3′ sequences of 120 nt. Final consensus full genomes had 10× coverage. We downloaded full-genome henipaviruses from GenBank, generated alignment by using the MAFFT plugin in Geneious, and tested node robustness by using 1,000 bootstrap replicates under the general time reversible plus gamma model in PHYML (*15*).

**Figure 2:**
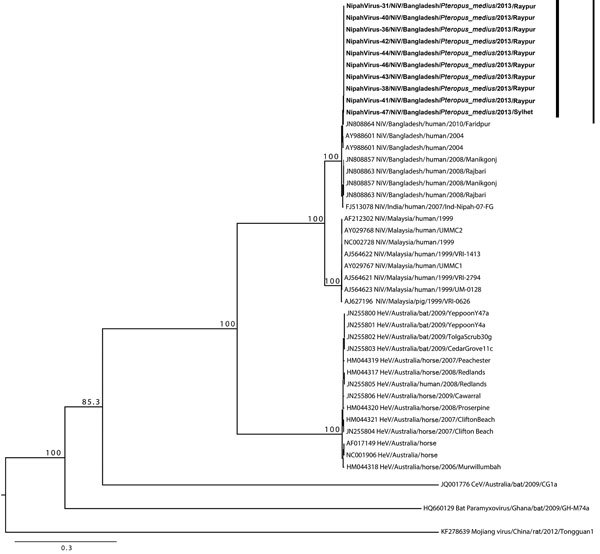
Phylogenetic tree of Nipah viruses from bats in Bangladesh (bold) compared with other henipaviruses, generated from full-genome sequences. Tree was constructed by using a maximum-likelihood approach, and robustness of nodes was tested with 1,000 bootstrap replicates. Sequences are labeled according to the following ordination: GenBank accession number or isolate identification number/virus type/country/host/year/strain. Numbers along branches are bootstrap values. Scale bar indicates nucleotide substitutions per site.

Full-length genome sequences were obtained from 10 bat NiV isolates. We constructed a phylogenetic tree (Figure 2) that showed all bat isolates had nearly identical genome sequences; there was 99.9% conservation among all 10 genomes characterized (Table). The virus isolated in Sylhet during January 2013 was nearly identical to all other isolates obtained in 2013 in Raipur, which is ≈350 km from Sylhet, suggesting that NiV homogeneity might be supported by bat movements connecting disparate bat colonies (*10*).

**Table:** Characteristics of 11 bat samples tested for Nipah virus, Bangladesh*.

Sample no.	Sample ID	Date sampled	Location	Sample type*	Taqman PCR C_t_	NGS	Isolate designation
1	PGB-1401B	2011 Apr 13	Raipur, Manikganj	ORU	31.9	Yes	NiV/BD/BA/2013/Raipur1401
2	PGB-1402B	2011 Apr 13	Raipur, Manikganj	ORU	30.1	Yes	NiV/BD/BA/2013/Raipur1402
3	PGB-1403B	2011 Apr 13	Raipur, Manikganj	ORU	31.0	Yes	NiV/BD/BA/2013/Raipur1403
4	PGB-1404B	2011 Apr 13	Raipur, Manikganj	ORU	31.3	Yes	NiV/BD/BA/2013/Raipur1404
5	PGB-1405B	2011 Apr 13	Raipur, Manikganj	ORU	31.0	Yes	NiV/BD/BA/2013/Raipur1405
6	PGB-1406B	2011 Apr 13	Raipur, Manikganj	ORU	31.9	Yes	NiV/BD/BA/2013/Raipur1406
7	PGB-1408B	2011 Apr 13	Raipur, Manikganj	ORU	31.3	Yes	NiV/BD/BA/2013/Raipur1408
8	PGB-1409B	2011 Apr 13	Raipur, Manikganj	ORU	31.7	Yes	NiV/BD/BA/2013/Raipur1409
9	PGB-1410B	2011 Apr 13	Raipur, Manikganj	ORU	31.0	Yes	NiV/BD/BA/2013/Raipur1410
10	PGB-1411B	2011 Apr 13	Raipur, Manikganj	ORU	30.8	No	NiV/BD/BA/2013/Raipur1411
11	PGB-191	2006 Jan 13	Sylhet	RU	39.0	Yes	NiV/BD/BA/2013/Sylhet191

*C_t_, cycle threshold; ID, identification; NGS, next-generation sequencing; NIV, Nipah virus; ORU, outbreak roost urine; RU, roost urine.

## Conclusions

We report isolation of NiV from *P. medius* bats, a natural virus reservoir in Bangladesh. With an improved enrichment-based NGS strategy, we generated complete genome sequences for 10 bat NiV isolates with higher efficiency than for traditional PCR-based sequencing methods. NiV has been difficult to isolate from bats and, similar to results of previous studies of Hendra virus (*10*), we observed that successful virus isolation does not correlate with cycle thresholds. The complete sequence identity match among isolates obtained during the outbreak investigation in Raipur suggests that multiple strains were not co-circulating in the bat population at the time, supporting results of a previous study in Faridpur (*10*).

None of the bat NiV isolate sequences were identical with any previously detected human NiV isolate sequences, suggesting that NiV spillover into humans is a rare event. However, the genetic diversity of bat NiV isolates needs to be fully identified.
